# Distinct Morphological, Physiological, and Biochemical Responses to Light Quality in Barley Leaves and Roots

**DOI:** 10.3389/fpls.2019.01026

**Published:** 2019-08-14

**Authors:** Karel Klem, Albert Gargallo-Garriga, Wutthida Rattanapichai, Michal Oravec, Petr Holub, Barbora Veselá, Jordi Sardans, Josep Peñuelas, Otmar Urban

**Affiliations:** ^1^Global Change Research Institute, Czech Academy of Sciences, Brno, Czechia; ^2^Centro de Investigación Ecológica y Aplicaciones Forestales (CREAF), Barcelona, Spain; ^3^Department of Soil Science, Kasetsart University, Bangkok, Thailand; ^4^Global Ecology Unit CREAF-CSIC-UAB, Barcelona, Spain

**Keywords:** light quality, photosynthesis, morphology, root system architecture, metabolomics

## Abstract

Light quality modulates plant growth, development, physiology, and metabolism through a series of photoreceptors perceiving light signal and related signaling pathways. Although the partial mechanisms of the responses to light quality are well understood, how plants orchestrate these impacts on the levels of above- and below-ground tissues and molecular, physiological, and morphological processes remains unclear. However, the re-allocation of plant resources can substantially adjust plant tolerance to stress conditions such as reduced water availability. In this study, we investigated in two spring barley genotypes the effect of ultraviolet-A (UV-A), blue, red, and far-red light on morphological, physiological, and metabolic responses in leaves and roots. The plants were grown in growth units where the root system develops on black filter paper, placed in growth chambers. While the growth of above-ground biomass and photosynthetic performance were enhanced mainly by the combined action of red, blue, far-red, and UV-A light, the root growth was stimulated particularly by supplementary far-red light to red light. Exposure of plants to the full light spectrum also stimulates the accumulation of numerous compounds related to stress tolerance such as proline, secondary metabolites with antioxidative functions or jasmonic acid. On the other hand, full light spectrum reduces the accumulation of abscisic acid, which is closely associated with stress responses. Addition of blue light induced accumulation of γ-aminobutyric acid (GABA), sorgolactone, or several secondary metabolites. Because these compounds play important roles as osmolytes, antioxidants, UV screening compounds, or growth regulators, the importance of light quality in stress tolerance is unequivocal.

## Introduction

Plants growing under natural conditions are exposed to light that varies in intensity, duration, and spectral quality due to changes in canopy filtering, cloud cover, and diurnal and seasonal variations. In addition, it can be challenging to determine the optimal light spectrum and intensity when cultivating plants under artificial conditions, such as in greenhouses or growth chambers (Ouzounis et al., 2015). Light-mediated photosynthesis is the predominant source of energy for plants, but light also provides important sensory signals for plants because it can promote acclimation to different environmental conditions, modulate growth and development, alter physiological functions, and regulate biochemical pathways (Kami et al., 2010).

Research during recent decades has identified three major groups of sensory photoreceptors in plants (Chen et al., 2004): the phytochromes (PHYs), which absorb light strongly in the red (600 to 700 nm) and far-red (700 to 800 nm) regions (Casal et al., 1998); the cryptochromes (CRYs), phototropins (PHOTs), and Zeitlupe family proteins, which absorb light strongly in the blue (400 to 500 nm) and ultraviolet-A (UV-A; 315 to 400 nm) regions (Galvão and Fankhauser, 2015); and UVB-RESISTANCE locus 8 (UVR8), which absorbs light strongly in the UV-B (280 to 315 nm) region (Tilbrook et al., 2013). In contrast to the many photobiology studies on above-ground plant organs, very few studies have investigated the role of light spectral quality on roots (Gundel et al., 2014). However, there is evidence that the red:far-red (R:FR) ratio mediates plant–plant interactions, in that it allows a plant to detect neighboring plants, and promotes rapid root growth and escape from the main zone of competition (Ballaré, 2009). Light spectral quality is also an important signal that allows plants to acclimate to changing environmental conditions, particularly abiotic stressors (Catalá et al., 2011). Light-induced metabolic reprogramming can lead to accumulation of anti-stress compounds, such as osmolytes, antioxidants, and stress-responsive proteins (Obata and Fernie, 2012).

Plant growth largely depends on the availability of carbohydrates synthesized during photosynthesis, and this is limited by numerous environmental factors in addition to light, such as temperature, water availability, nutrient availability, and pathogens (Kangasjärvi et al., 2012). The sugar-mediated regulation of plant growth interacts with multiple phytohormones (Das et al., 2012) and the circadian clock (Lastdrager et al., 2014), and both are connected to light signaling (de Lucas and Prat, 2014). Thus, it is likely that crosstalk between sugar production and light signaling also regulates plant growth. Monochromatic red light stimulates the accumulation of glucose and fructose, while combined red and blue light significantly enhances the accumulation of sucrose and starch (Li et al., 2017). Such changes are mainly associated with activities of invertases and explain also the effects on photosynthetic performance and plant morphology. Sugar, and particularly sucrose allocation, plays a crucial role in long-distance communications between shoot and root and coupling of carbon and nitrogen metabolisms involving cytokinin, auxin, and small peptide signals (Wang and Ruan, 2016). Since the phosphorylated hexoses are required in different metabolic processes and plant development, hexose-phosphorylating enzymes (mainly hexokinase) are essential in sugar sensing and orchestration of photosynthetic carbon fixation and water and nutrient uptake (Granot et al., 2014). Light intensity and daytime have a major effect on sugar transport in the phloem, but it was significantly affected also by light quality at the medium photosynthetic rates (Lanoue et al., 2018). Sugar-mediated regulation of plant growth and photosynthesis can be thus attributed to the effects of both light intensity and quality.

Besides the effects of light on primary metabolism, its spectral quality also influences the biosynthesis of secondary metabolites (particularly carotenoids, phenolic compounds, and anthocyanins) that function as antioxidants or UV screening compounds (Bian et al., 2015). UV and blue light (perceived by UVR8 and CRYs) affect the biosynthesis of numerous secondary metabolites including phenolic acids (e.g., chlorogenic acid, *p*-coumaric acid, caffeic acid), flavonoids (e.g., kaempferol, quercetin, rutin, anthocyanin), and carotenoids (lutein, violaxanthin, zeaxanthin, neoxanthin, β-carotene; reviewed by Ouzounis et al., 2015). These compounds, in turn, enhance the photoprotective capacity of plants against high radiation stress (Klem et al., 2015). Although the transition between regulatory effect and stress response to light intensity and quality is gradual (Robson et al., 2015), particularly at high intensities of UV-B radiation, the stress responses involving DNA damage, lipid peroxidation, photoinhibition, and/or growth inhibition often occur in plants (Hideg et al., 2013).

Based on a review of the literature, Gundel et al. (2014) suggested that light spectral quality considerably affects root growth, morphology, and functional interactions with soil microorganisms. Light spectral quality can thus play an important ecological role by altering root–shoot interactions. For instance, Sadras et al. (1989) showed that the roots of crops grown at high density penetrate deeper soil layers than crops grown at low density because of differences in the above-ground R:FR ratio. Light spectral quality can therefore greatly impact the efficiency of the uptake of resources (water and nutrients) from the soil and affect the way the plants adjust to competition for soil resources. However, a meta-analysis by Poorter et al. (2012) found that the response of roots to the R:FR ratio depended on plant ontogeny (Green-Tracewicz et al., 2012), intensity of light (McLaren and Smith, 1978), proximity of other plants (Murphy and Dudley, 2009), and nutrient availability (Aphalo and Lehto, 2001). Gundel et al. (2014) proposed that the R:FR ratio affects root system architecture (RSA) by altering the levels of the plant hormone auxin. Auxin is a well-known regulator of root development (Blilou et al., 2005) and architecture (Péret et al., 2009) and can also induce directional root growth away from potential competitors.

The blue and UV light photoreceptors (CRYs, PHOTs, and UVR8) also alter signaling pathways connected to the phytohormone-mediated regulation of growth, development, physiological functions, and biochemical pathways, so it seems likely that they also affect RSA (Gelderen van et al., 2018a). Photoreceptors have important roles in plant resource management because they control the allocation of resources needed for growth and the transition of metabolism between growth and stress-coping states based on light availability. Thus, crop scientists have a renewed interest in photoreceptors because of their effect on root–shoot interactions and the possible implications of these responses to economically important crops.

Besides the direct effect of light intensity and quality on balancing plant growth and defense mechanisms, the circadian clock and redox rhythm serve also as an important regulatory mechanism. Such mechanisms involve especially biosynthesis of the major antioxidant (glutathione) and plant defense hormones [jasmonic acid (JA) and salicylic acid], thus adjusting plant immunity (Karapetyan and Dong, 2018).

The main aim of this study was to test the hypothesis that light spectral quality changes the allocation of plant biomass between above-ground parts and roots, alters the RSA, affects interactions among neighboring plants, and impacts the ability of plants to cope with reduced availability of water or nutrients. The effect of spectral quality was tested on two barley varieties. Barke variety is considered as highly sensitive to oxidative stress, while the variety Bojos is commonly grown in Central European conditions without symptoms of oxidative stress damage. We also hypothesized that light spectral quality affects the accumulation and allocation of metabolites involved in stress tolerance, such as plant hormones, antioxidants, and osmolytes, and thereby modulates tolerances to different stressors.

## Material and Methods

### Plant Material and Experimental Design

Seeds of spring barley (*Hordeum vulgare*) varieties Barke (sensitive to oxidative stress; Klem et al., 2012) and Bojos (tolerant to oxidative stress) were provided by the barley gene bank of the Agricultural Research Institute Kroměříž Ltd., Czech Republic. Seeds were germinated on moistened germination paper for 48 h at 26°C in the dark. Germinating seeds were then transplanted into the growth unit, in which the roots grew on black filter paper that was between two black plastic sheets, and a micro-irrigation system recirculated a nutrient solution through the roots (**Figure S1**; for details, see Rattanapichai and Klem, 2016). The nutrient solution was Hoagland’s No. 2 basal salt mixture (Sigma Aldrich Chemie, Steinheim, DE; concentration: 1.6 g L^−1^, pH range: 4.5 to 5.2). The solution was replaced every 7 days throughout the 26-day experiments.

The growth units were placed in five large growth chambers (FS-SI-3400, PSI, Drásov, CZ), which allowed continuous regulation of light intensity in the red, blue, far-red, and UV-A spectral regions. Light-emitting diodes (LEDs) were the source for radiation in the red (R; maximum emission: 640 nm), blue (B; maximum emission: 450 nm), and far-red (FR; maximum emission: 740 nm) regions, and fluorescent lamps (LT 30W T8/010UV, Narva, DE) were the source for UV-A radiation (maximum emission: 370 nm). The following five light regimens were used: **R**, R light only; **R-B**, R and B lights at 1:1 ratio of photon flux density; **R-FR**, R and FR lights; **R-UVA**, R and UV-A lights; and **R-B-FR-UVA**, R and B radiation in a 1:1 ratio of photon flux density with additional FR and UV-A light. The intensity of photosynthetically active radiation (PAR, 400 to 700 nm) was the same for all treatments (130 μmol photons m^−2^ s^−1^). The intensity of UV-A was set to 4 W m^−2^, and the intensity of FR light was adjusted to provide a 0.6 ratio of R:FR. Each light regimen and barley variety had four replicates (four separate growth units), which were randomized within each growth chamber. The growth units and plants were transferred between growth chambers while maintaining the same light regimen, and newly randomized every 7 days to avoid possible artifacts from individual growth chambers. All plants were grown under a 12/12 h day/night regimen, with a day/night temperature of 25/20°C and a day/night relative humidity of 60%/80%. Photon flux density in the range of 400–700 nm (PAR) was measured using light meter Li-250 (LI-COR Biosciences, Lincoln, NE, US) with quantum sensor Li-190 (LI-COR Biosciences). The emission spectra of LEDs and fluorescent lamps were measured using spectroradiometer AvaSpec-2048-USB2 (Avantes BV, Apeldoorn, NL), with grating and a measurement range of 200–1100 nm. R:FR ratio was calculated by integration photon flux density between 655–665 nm UV/VIS/NIR (R) and 725–735 nm (FR).

### Root and Shoot Morphology

Roots and shoots were scanned with a large area scanner (LA2400, Regent Instruments, Quebec, CA) at a resolution of 300 pixels per inch. Total (projected) leaf area (TLA) and leaf length were then measured using ImageJ software. For subsequent statistical analyses, TLA and length of the youngest completely developed leaf (4^th^ leaf) were determined because these were most sensitive to light regimens.

The RSA parameters were evaluated using WinRHIZO software (Regent Instruments). The parameters measured were: total root length (TRL), total seminal root length (TSL), total lateral root length (TLL), total root surface area (TSA), and average branching angle (BA). Plant material was subsequently used for metabolomic analyses.

### Plant Physiology

All physiological measurements were conducted between 10:00 and 14:00 Central European Time (CET). Basic photosynthetic parameters were measured on the 4^th^ leaf using a Li-6400 gas-exchange system (LI-COR Biosciences) at a CO_2_ concentration of 400 μmol CO_2_ mol^−1^. During these measurements, leaf temperature (25°C) and humidity (60%) were kept constant in the growth chamber. Light-saturated rates of the following photosynthetic parameters were measured at a photosynthetic photon flux density of 1200 μmol photons m^−2^ s^−1^ after 3 to 5 min of acclimation: light-saturated CO_2_ assimilation rate, *A*_max_; light-saturated stomatal conductance, *G*_Smax_; and light-saturated transpiration rate, Tr.

Chlorophyll *a* fluorescence (Chl-F) parameters were estimated on the light-adapted 4^th^ leaves using a pulse amplitude modulated fluorometer (PAM 2500, H. Walz, Effeltrich, DE). Before these measurements, the leaves were acclimated to a photosynthetic photon flux density of 130 μmol photons m^−2^ s^−1^ for 3 min, and the same photon flux density of actinic light was used for the measurement. The Chl-F signal at the red band (near 690 nm) was measured using short flashes (10 ms pulses, intensity approximately 0.003 μmol photons m^−2^ s^−1^) that were 800 ms apart (F_S_). Maximum Chl-F (F_M_′) was recorded during 1 s application of a saturating light pulse (approx. 5,000 μmol photons m^−2^ s^−1^). Minimum Chl-F in the light-adapted state (F_0_′) was estimated after a saturating pulse when the actinic light was off, and the FR light was on for 5 s. The actual quantum yield of photosystem (PS) II photochemistry [Φ_PSII_ = (F_M_′ – F_S_)/F_M_′] and thermal energy dissipation [D = 1 – (F_M_′ – F_0_′)/F_M_′] were calculated according to Demmig-Adams et al. (1996).

Chlorophyll and epidermal flavonols were estimated indirectly as chlorophyll and flavonol indices, based on light transmittance and UV screening of Chl-F (Dualex Flav, Force A, Orsay, FR). Two measurements in the central part of the 4^th^ leaf were performed.

### Metabolomic Analyses

The upper three leaves (2^nd^–4^th^ leaf) and roots from each replicate were sampled between 11:00 and 14:00 (CET) and immediately frozen in liquid nitrogen for metabolomic analyses. The samples were homogenized using a mortar and pestle with liquid nitrogen and then extracted using methanol:chloroform:H_2_O solution (1:2:2). An aliquot of the upper (polar) phase was used to analyze metabolites using an UltiMate 3000 high-performance liquid chromatograph (HPLC) (Thermo Fisher Scientific, US/Dionex RSLC, Dionex, US) coupled with an LTQ Orbitrap XL high resolution mass spectrometer (HRMS) (Orbitrap, Thermo Fisher Scientific, Waltham, MA, US) that was equipped with a heated electrospray ionization source. All samples were analyzed in the positive and negative polarity of Orbitrap, operated in full-scan mode over a range of m/z 50 to 1,000 (positive mode) and 65 to 1,000 (negative mode).

A Hypersil Gold column (C18; 150 mm × 2.1 mm, 3 μm; Thermo Fisher Scientific, US) was used for chromatographic separation. The flow rate of the mobile phase was 0.3 ml min^−1^, and the column temperature was 30°C. The mobile phase consisted of A) acetonitrile and B) water with 0.1% acetic acid. Both mobile phases (A and B) were filtered and degassed for 10 min in an ultrasonic bath before use. Gradient elution chromatography was performed starting with 10% A and 90% B for 5 min. From 5 to 20 min, the proportion of A increased steadily to 90% as B declined to 10%, and this condition was maintained for 5 min. Then, the system was equilibrated to the initial condition (10% A and 90% B) over 5 min. The absorbances at 254, 272, 274, and 331 nm were monitored. Acetonitrile hyper-grade for liquid chromatography - mass spectrometry (LC-MS) LiChrosolvR was from Merck KgaA (Darmstadt, DE), acetic acid was from Sigma Aldrich Chemie, and the Purelab Classic (ELGA LabWater, High Wycombe, Bucks, UK) was used to generate high-purity water for preparation of the aqueous mobile phase.

First, all metabolites were identified using a standard library (>200 compounds) and confirmed by mass, retention time (RT), isotopic pattern, and ring double-bon+d parameters. Then, identifications were confirmed by comparing chromatogram peaks with the KEGG database (Kanehisa and Goto, 2000) using a built-in M/Z MINE utility with a 5 ppm threshold. The third approach of metabolite identification used the MASSBANK databases (Horai et al., 2010), with searches for each mass spectra (MS) and tandem mass spectra (MS/MS). We tentatively assigned metabolites to molecular ions and exact masses corresponding to metabolites in these databases. Data, including detailed interpretations of the MS/MS spectra that supported our tentative identifications of masses, are referred to as not positively identified throughout the manuscript. The raw data from the HPLC-HRMS system were processed and compared using the XCMS processing platform for peak detection and matching (Smith et al., 2006).

### Statistical and Bioinformatic Analyses

Morphological and physiological data were analyzed using a two-way fixed-effect analysis of variance (ANOVA) model using Statistica 12 software (StatSoft, Tulsa, US). Tukey’s *post hoc* test (*p* = 0.05) was used to identify significant differences between the means.

The peak area corresponding to each metabolite was normalized based on the total peak area in the sample. Neutral masses obtained in positive and negative modes were evaluated to avoid duplicates (same RT and neutral mass in different modes) and to retain the greatest number and largest peaks. The differences of the metabolomes of each barley variety that received different light regimens were identified by a permutational analysis of variance (PERMANOVA) (Anderson et al., 2008) of the HPLC–HRMS data for each replication using Euclidean distances, in which light regimen and variety were the fixed factors, and there were 2000 permutations. We also used principal component analysis (PCA) to determine the relationships of light regimen, barley variety, and root and shot metabolites most responsible for these differences between different levels of experimental factors. The PCA was performed using R v2.12 Core functions.

## Results

### Leaf Area and Length

**Figure 1:**
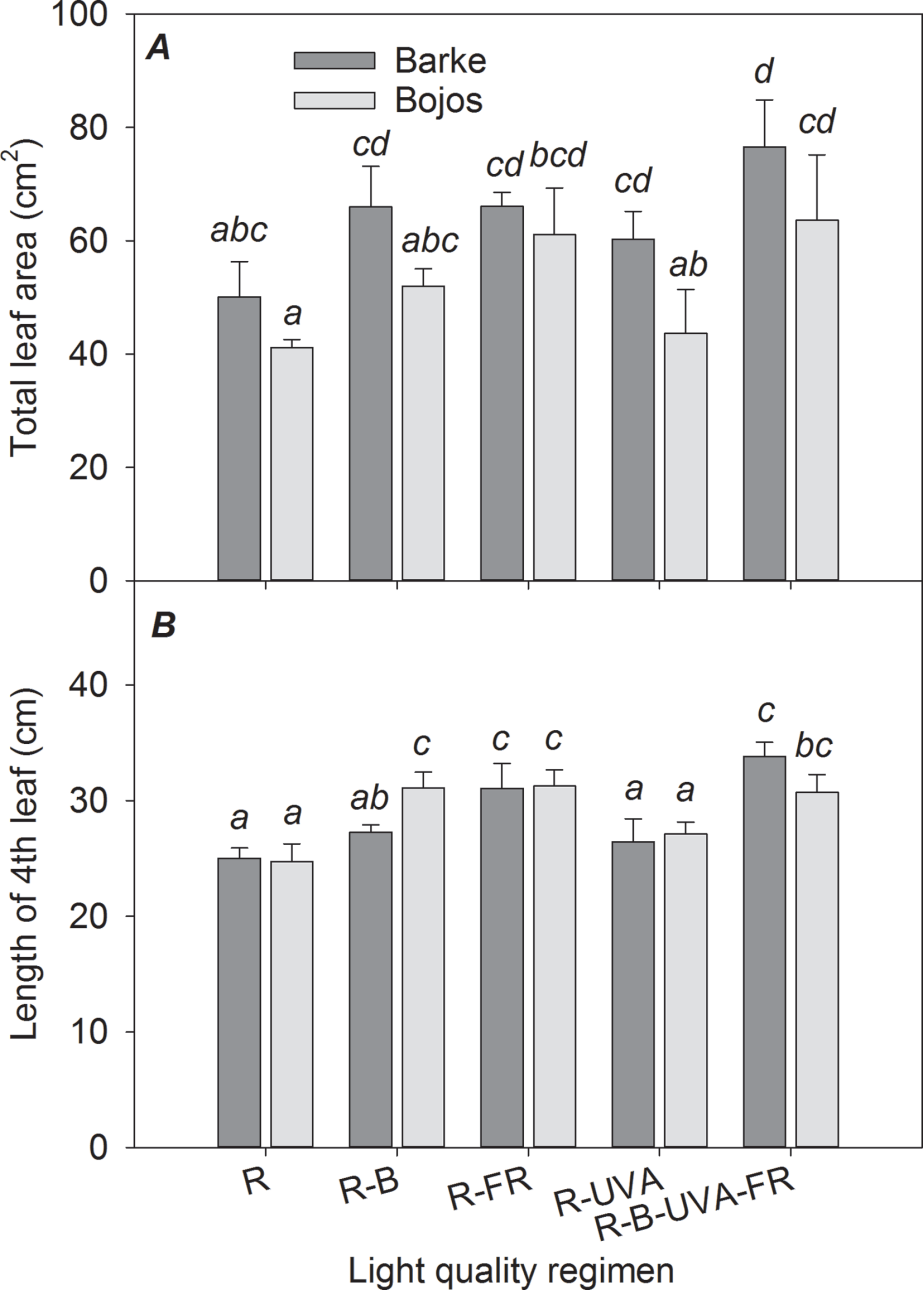
Effect of 26 days exposure to different light regimens on total leaf area per plant **(A)** and length of the last completely developed (4th) leaf **(B)** of spring barley varieties Barke (dark grey) and Bojos (light grey). Here and in subsequent figures: R, only red radiation; R-B, red and blue radiation (1:1 ratio of photon flux density); R-FR, red and far-red radiation; R-UVA, R and UV-A radiation; R-B-FR-UVA, red and blue radiation (1:1 ratio of photon flux density) with far-red and UV-A radiation. All treatments used a photosynthetic photon flux density of 130 µmol m^−2^ s^−1^. Column height indicates mean, vertical bars indicate standard deviations (*n* = 4), and different letters indicate significant differences (*p* ≤ 0.05) based on Tukey’s ANOVA post hoc test.

The Barke variety generally had a greater TLA than the Bojos variety by 23%, but this difference was only significant for the R-UVA regimen (**Figure 1A**). The R-B, R-FR, and R-UVA regimens led to greater TLA than the R regimen (by 29%, 40%, and 13%, respectively), but these differences were not statistically significant for the R-B and R-UVA regimens. Relative to the R regimen, the R-B-UVA-FR regimen significantly increased TLA in both varieties (by 54%), but the R-FR regimen significantly increased the TLA only in the Bojos variety. Generally, the R-B-UVA-FR regimen led to the greatest TLA. The length of the 4^th^ leaf was similar for the different light regimens, and there were no differences between varieties (**Figure 1B**). Relative to the R regimen, the R-FR and R-B-FR-UVA regimens led to significantly greater length of the 4^th^ leaf in both varieties (by 25% and 30%, respectively). The length of the 4^th^ leaf was also significantly greater under the R-B regimen than under the R regimen in the Bojos variety (by 26%).

### Root System Architecture

**Figure 2:**
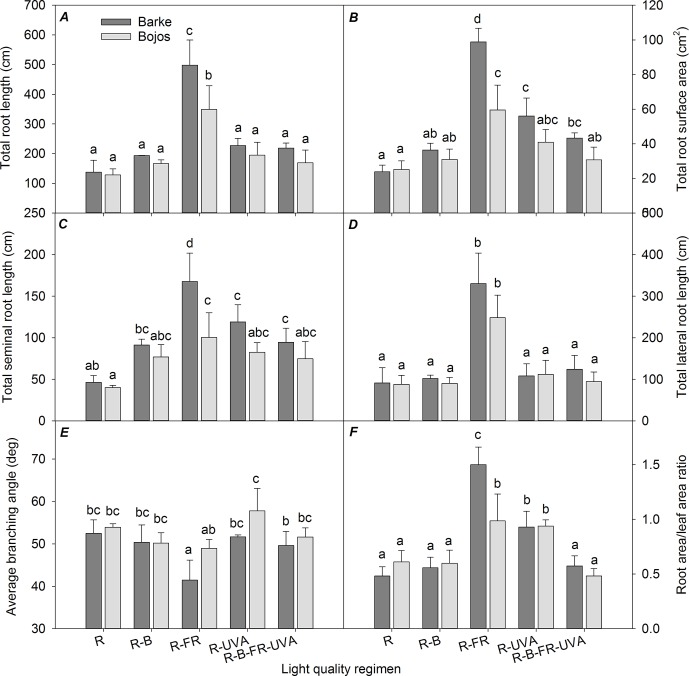
Effect of 26 days exposure to different light regimens on total root length **(A)**, total root surface area **(B)**, total seminal root length **(C)**, total lateral root length **(D)**, average branching angle **(E)**, and ratio of total root area:total leaf area **(F)** of spring barley varieties Barke (dark grey) and Bojos (light grey). Additional details are in the **Figure 1** legend.

Similar to the TLA results, TSA, TRL, and lengths of the seminal and lateral roots (TSL and TLL) were greater in the Barke variety than the Bojos variety under most light regimens (by 42%, 32%, 41%, and 26%, respectively; **Figures 2A, B, C, D**); however, these varieties only had statistically significant differences in TSA, TRL, and TSL for the R-FR regimen. Relative to the R regimen, the R-FR regimen significantly increased TRL (by 217%), TSA (by 225%), TSL (by 206%), and TLL (by 222%) in both varieties, but this regimen significantly decreased the BA in the Barke variety (by 21%) (**Figure 2E**). Also, relative to the R regimen, the R-UVA and R-B-UVA-FR regimens substantially increased TRL (by 65% and 59%, respectively), TSA (by 135% and 81%, respectively), and TSL (by 157% and 104%, respectively) in the Barke variety, although the effects were statistically insignificant in the Bojos variety.

The TSA : TLA ratio was significantly greater under the R-FR (by 136%) and R-UVA (by 73%) regimens than the R regimen. The effect of the R-FR regimen on this ratio was only significantly greater that of the R-UVA in the Barke variety (**Figure 2F**).

### Chlorophyll and Epidermal Flavonols

**Figure 3:**
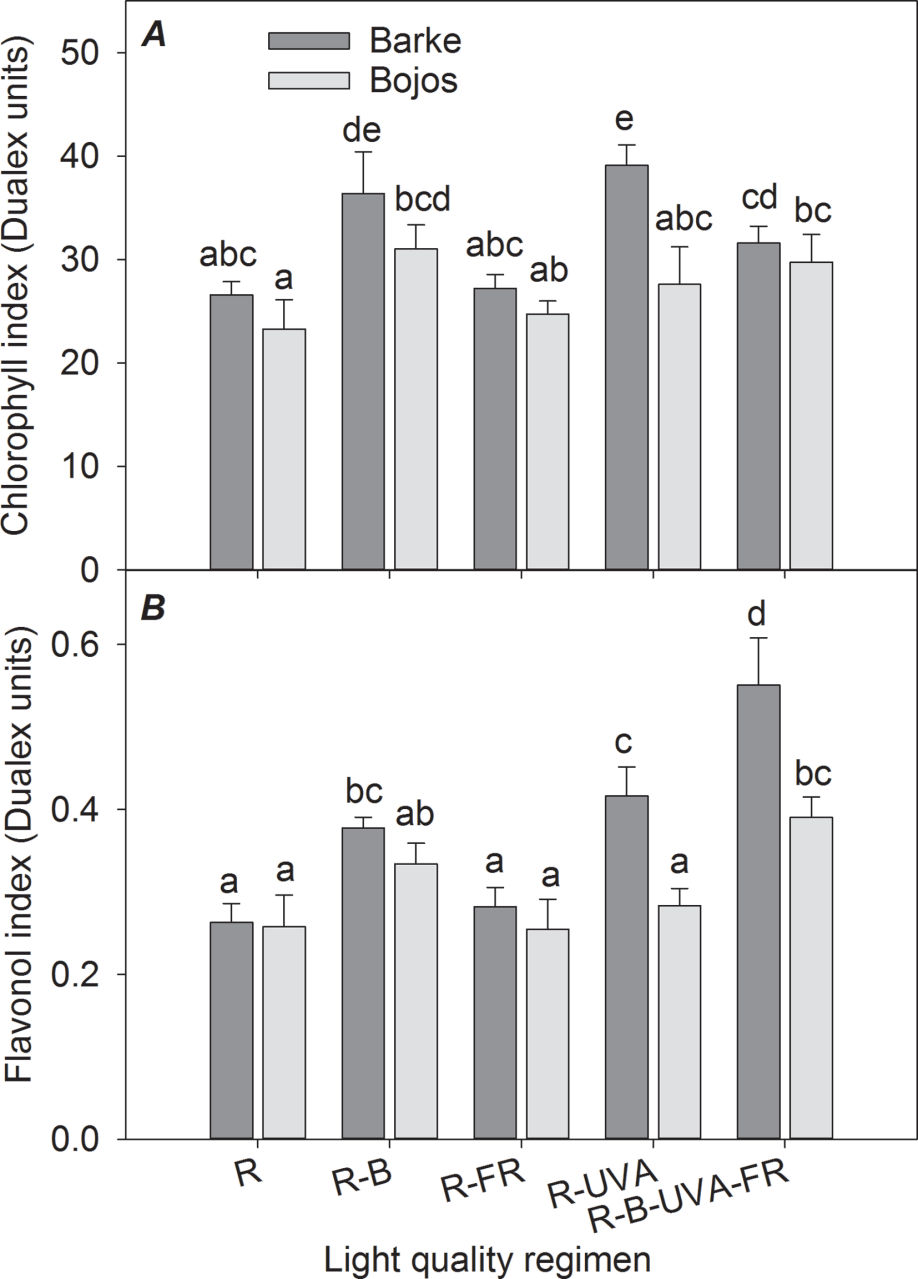
Effect of 26 days exposure to different light regimens on chlorophyll index **(A)** and flavonol index **(B)** of spring barley varieties Barke (dark grey) and Bojos (light grey). Additional details are in the **Figure 1** legend.

The chlorophyll index (**Figure 3A**) was greater under the R-B regimen than the R regimen in both varieties (by 35%). Furthermore, relative to the R regimen, the R-UVA regimen led to a greater chlorophyll index in the Barke variety (by 47%), and the R-B-UVA-FR regimen led to a greater chlorophyll index in the Bojos variety (by 28%). The light regimen had a stronger effect on the flavonol index (**Figure 3B**) in the Barke variety than the Bojos variety. Relative to the R regimen, the flavonol index was only greater in the Bojos variety under the R-B-UVA-FR regimen (by 51%); the R-B-UVA-FR, R-UVA, and R-B regimens increased the flavonol indexes in the Barke variety (by 110%, 58%, and 44%, respectively). Relative to the R regimen, the R-B-UVA-FR regimen led to the greatest increase of the flavonol index.

### Photosynthetic Parameters

**Figure 4:**
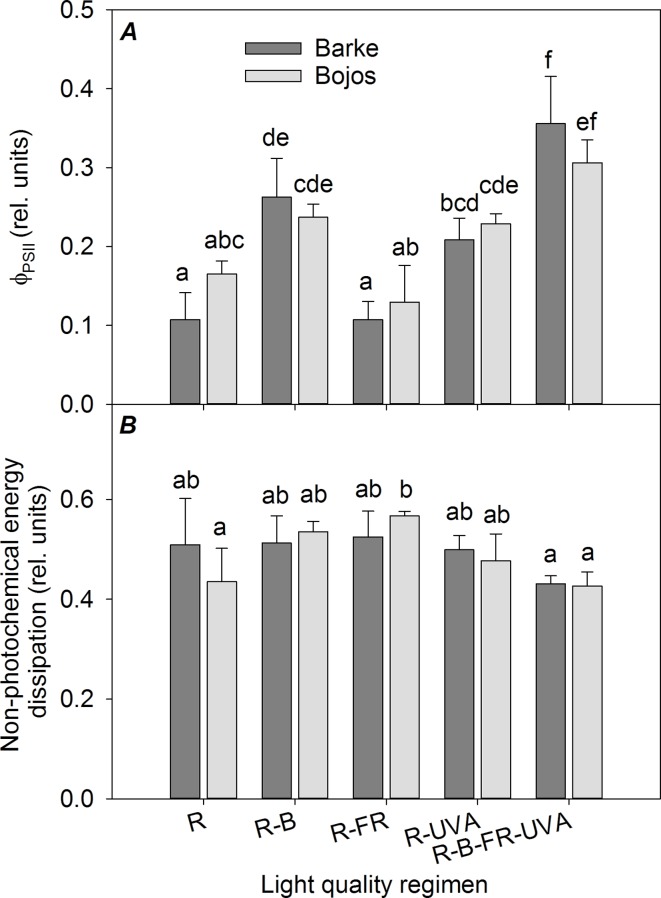
Effect of 26 days exposure to different light regimens on actual quantum yield of PSII photochemistry (Φ_PSII_, **A**) and non-photochemical energy dissipation **(B)** of spring barley varieties Barke (dark grey) and Bojos (light grey). Additional details are in the **Figure 1** legend.

The actual quantum yield of PSII photochemistry (F_PSII_) was significantly greater in the Barke variety under the R-UVA (by 94%), R-B (by 145%), and R-B-UVA-FR (by 231%) regimens relative to the R regimen (**Figure 4A**). The results were similar but less pronounced in the Bojos variety (by 39%, 44%, and 85%, respectively); the only significant increase in this variety was under the R-B-UVA-FR regimen. The effects of both light regimens on non-photochemical energy dissipation were generally small and statistically insignificant in both varieties (**Figure 4B**).

**Figure 5:**
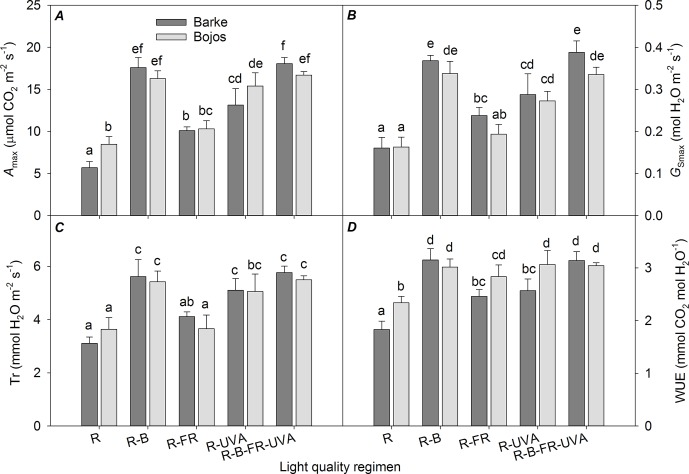
Effect of 26 days exposure to different light regimens on light-saturated CO_2_ assimilation rate (*A*_max_, **A**), light-saturated stomatal conductance (*G*_Smax_, **B**), transpiration rate (Tr, **C**), and water use efficiency (WUE, **D**) of spring barley varieties Barke (dark grey) and Bojos (light grey). Additional details are in the **Figure 1** legend.

The light-saturated rates of CO_2_ assimilation (*A*_max_), transpiration (*Tr*_max_), and stomatal conductance (*G*_Smax_) were similar to the F_PSII_ results (**Figures 5A, B, C**). In both varieties, these parameters were significantly increased under the R-B (by 151%, 65%, and 118%, respectively), R-UVA (by 106%, 52%, and 73%, respectively), and R-B-UVA-FR (by 157%, 68%, and 124%, respectively) regimens relative to the R regimen. In addition, the R-FR regimen significantly increased the *A*_max_ and *G*_Smax_ in the Barke variety (by 77% and 48%, respectively). Water use efficiency (ratio of *A*_max_ and *Tr*_max_) was significantly less under the R regimen than under the other four regimens (the regimens with the addition of B, FR, and UV-A increased water use efficiency by 28–51%; **Figure 5D**).

### Metabolite Profiling

Overall, we detected 1,785 metabolites and identified 73 of them. Among the identified metabolites, amino acids were the most abundant, followed by carbohydrates, organic acids, and secondary metabolites. When the entire metabolomic data set was analyzed jointly for leaves and roots using PERMANOVA, the plant organ (root *vs.* shoot) explained almost half of the variance (pseudo-F = 49.03, p < 0.001; **Table 1**). The light regimen explained less of the total variance, but its effect was also significant (pseudo-F = 3.26, p < 0.001). Barley variety had a significant but smaller effect on the metabolite profile (pseudo-F = 2.19, p < 0.05). Analysis of all interactions indicated that the only significant interaction was between light regimen and plant organ (pseudo-F = 2.57, p < 0.05).

**Table 1:** PERMANOVA results for the entire metabolomic data set.

	Df	SS	MS	Pseudo-F	R^2^	P(perm)	
Barley variety	1	0.1818	0.1818	2.189	0.01551	0.05	*
Light regimen (Light)	4	1.085	0.2713	3.265	0.09256	0.001	***
Plant organ	1	4.0714	4.0714	49.013	0.34734	0.001	***
Variety:Light	4	0.2971	0.0743	0.894	0.02535	0.582	
Variety:Organ	1	0.1722	0.1722	2.073	0.01469	0.054	.
Light:Organ	4	0.8539	0.2135	2.57	0.07285	0.002	**
Variety:Light:Organ	4	0.3254	0.0814	0.979	0.02776	0.484	
Residuals	57	4.7348	0.0831		0.40394		
Total	76	11.7217			1		

*P(perm) 0.05>=0.01, **P(perm) 0.01>=0.001, ***P(perm) <=0.001.

**Figure 6:**
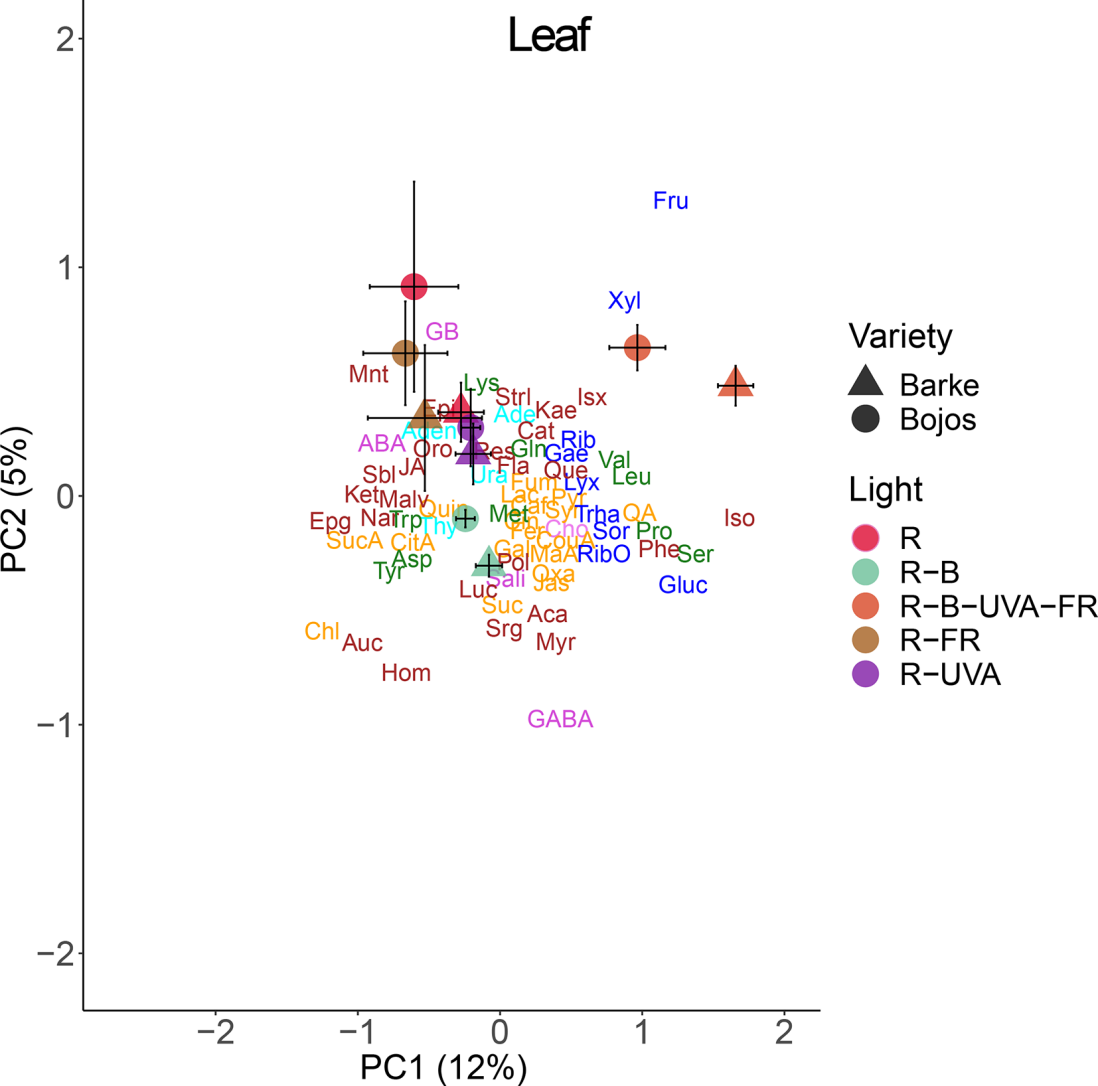
Principal component analysis (PC1 *vs.* PC2) of leaf metabolite concentrations of two barley varieties grown for 26 days under different light regimens. Here and in **Figures 8** and **9**: values for each variety (circles, Bojos; triangles, Barke) and light regimen (red, R; R-B, cyan; R-B-UVA-FR, orange; R-FR, brown; R-UVA, purple) are presented as means and standard deviations (error bars). Loadings of PC1 and PC2 for individual metabolite concentrations are represented by abbreviations. The data from individual replicates (*n* = 4) were used for the analysis. Biochemical families are represented by colors: dark blue, sugars; green, amino acids; yellow, compounds involved in the metabolism of amino acids and sugars; cyan, nucleotides; brown, phenolics; and violet, others. Abbreviations of metabolites: uracil (Ura), adenine (Ade), thymidine (Thy), adenosine (Aden), D-ribose (Rib), D-lyxose (Lyx), D-xylose (Xyl), D-fructose (Fru), 5-methylthio-D-ribose (RibO), D-sorbose (Sor), D-glucose (Gluc), D-galactose (Gae), D-(+)-trehalose dehydrate (Trha), lysine (Lys), glutamine (Gln), leucine (Leu), isoleucine (Iso), phenylalanine (Phe), methionine (Met), tryptophan (Trp), valine (Val), acid aspartic (Asp), tyrosine (Tyr), proline (Pro), serine (Ser), succinic acid (SucA), citrate acid (CitA), malic acid (Mal), lactic acid (Lac), quinic acid (Qui), pyruvate (Pyr), *p*-coumaric acid (CouA), jasmone (Jas), gallic acid (Gal), maleic acid (MaA), fumarate (Fum), trans-caffeic acid (Caf), aconitate (Suc), quinic acid (QA), trans-ferulic acid (Fer), syringic acid (Syr), oxaloglutarate (Oxa), jasmonic acid (JA), cinnamic acid (Cin), chlorogenic acid (Chl), γ-aminobutyric acid (GABA), glycine-betaine (GB), choline (Cho), sodium salicylate (Sali), (-)-abscisic acid (ABA), resveratrol (Res), acacetin (Aca), kaempferol (Kae), epicatechin (Epi), alpha-ketoglutaric acid (Ket), quercetin (Que), epigallocatechin (Epg), myricetin (Myr), aucubin (Auc), isovitexin (Isx), homoorientin (Hom), lucenin (Luc), malvidin (Malv), naringenin (Nar), flavan-3-ol (Fla), catechin (Cat), sorgolactone (Srg), (+)-orobanchol (Oro), (+)-strigol (Strl), polyols (Pol), mannitol (Mnt), sorbitol (Sbl).

A PCA analysis of metabolites in barley leaves also indicated a major effect of light regimen and small differences between varieties (**Figure 6**). PC1 mainly separated the effect of the R-B-UVA-FR regimen from other regimens, and PC2 showed the dominant effect of the R-B regimen. The R-B-UVA-FR regimen was associated with an accumulation of D-xylose and D-fructose and some secondary metabolites (isovitexin or kaempferol). The R, R-FR, and R-UVA regimens led to the accumulation of glycine-betaine, abscisic acid (ABA), mannitol, and ferulic acid. On the other hand, the R-B regimen led to the accumulation of γ-aminobutyric acid (GABA), sorgolactone, and several secondary metabolites (homoorientin, myricetin, aucubin, and acacetin). Analysis of barley variety indicated greater accumulation of glucose, serine, proline, isoleucine, and phenylalanine in the Barke variety than the Bojos variety.

**Figure 7:**
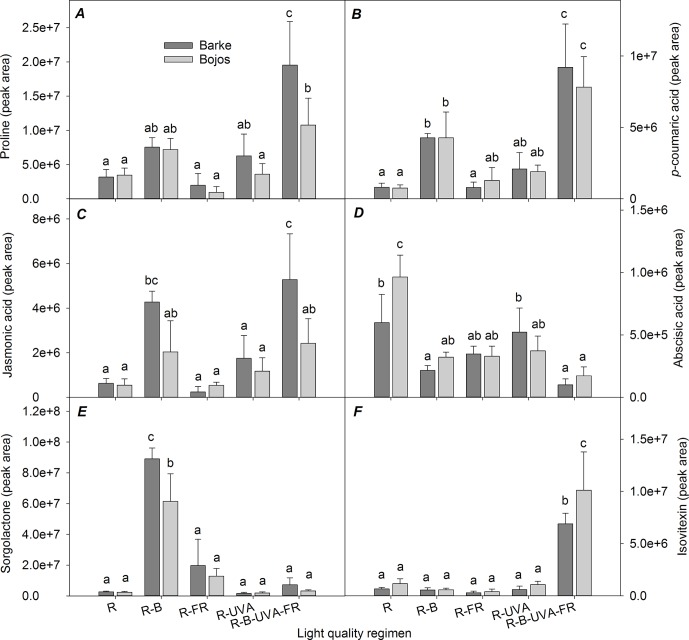
Effects of 26 days exposure to different light regimens on the levels of a representative amino acid (proline, **A**), plant hormones or compounds with regulatory effects (jasmonic acid, **C**; abscisic acid, **D**; sorgolactone, **E**), and phenolic compounds (*p*-coumaric acid, **B**; isovitexin, **F**) in leaves of spring barley varieties Barke (dark grey) and Bojos (light grey). Additional details are in the **Figure 1** legend.

**Figure 7** shows selected metabolites in barley leaves that had large differences among the different light regimens. These results show that there were high levels of proline and *p*-coumaric acid under the R-B-UVA-FR (by 365% and 983% relative to the R regimen) and R-B regimens (by 123% and 445% relative to the R regimen); however, the R-B regimen only had a significant effect on *p*-coumaric acid (**Figures 7A, B**).

The Barke variety was generally more sensitive to the R-B-UVA-FR regimen than the Bojos variety, in terms of proline (by 516% in Barke and 211% in Bojos relative to the R regimen) and JA (by 741% in Barke and 346% in Bojos relative to the R regimen) accumulation (**Figures 7A, C**), but the Barke variety accumulated less ABA (by 19% relative to Bojos; **Figure 7D**). Analysis of sorgolactone indicated increased accumulation under the R-B regimen relative to the R regimen (by 2,818%), but the R-B-UVA-FR regimen only had a weak effect (by 103%; **Figure 7E**). On the contrary, relative to the R regimen, the R-B-UVA-FR regimen led to significant accumulation of the flavonoid isovitexin (by 847%; **Figure 7F**).

**Figure 8:**
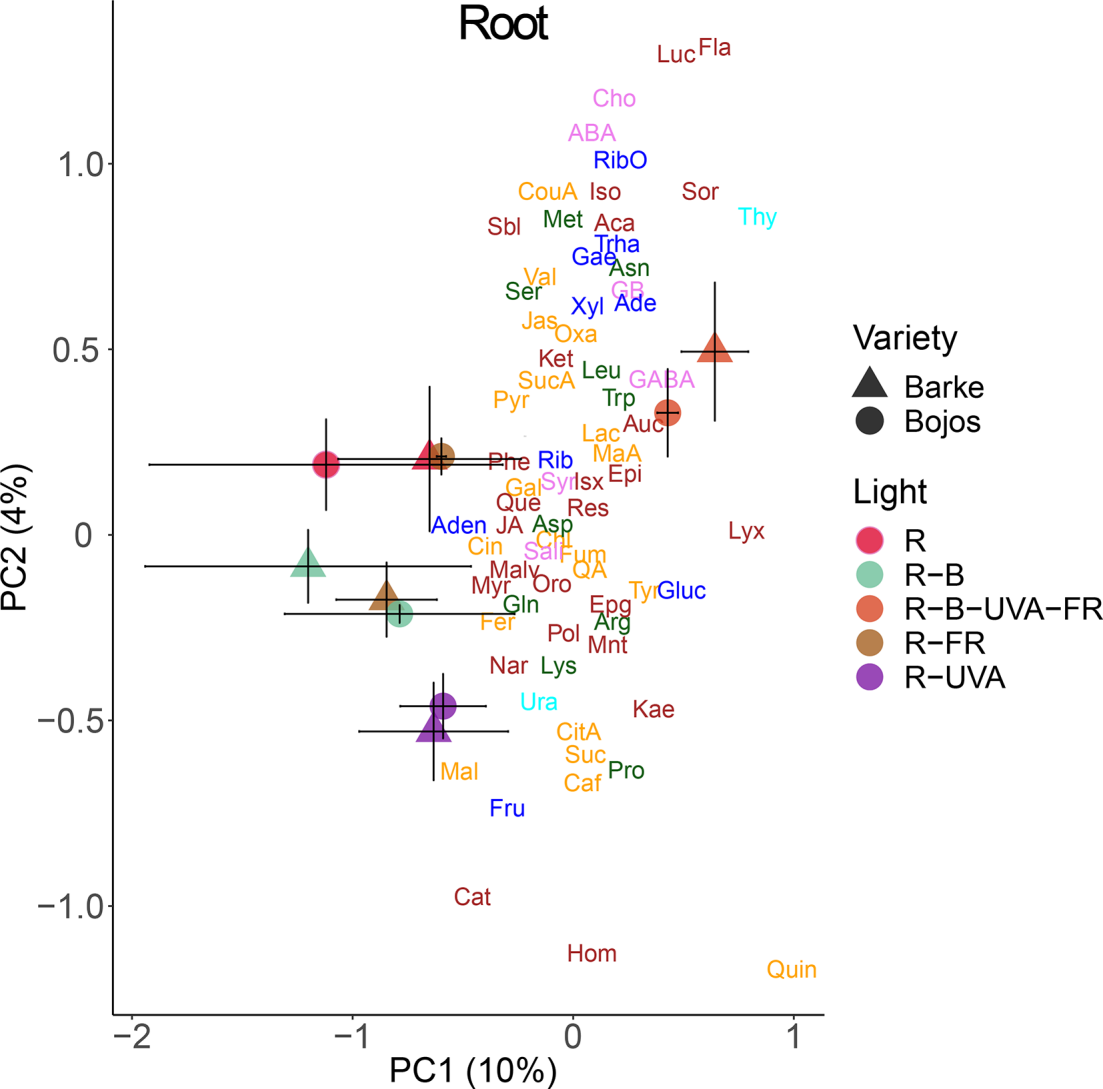
Principal component analysis (PC1 *vs.* PC2) of root metabolite concentrations of two barley varieties grown for 26 days under different light regimens. Additional details are in the **Figure 6** legend.

Analysis of roots indicated that they had substantially lower levels of metabolites than leaves. A PCA analysis indicated a small effect of barley variety on root metabolites (**Figure 8**). In particular, PC1 separated the effect of the R-B-UVA-FR regimen from the other regimens, and PC2 mainly separated the effects of the R-B and R-UVA regimens. The R-B-UVA-FR regimen was associated with greater levels of thymidine, D-sorbose, GABA, aucubin, and D-lyxose, but the addition of UV-A radiation increased the accumulation of L-malic acid, fructose, and acacetin in roots.

**Figure 9:**
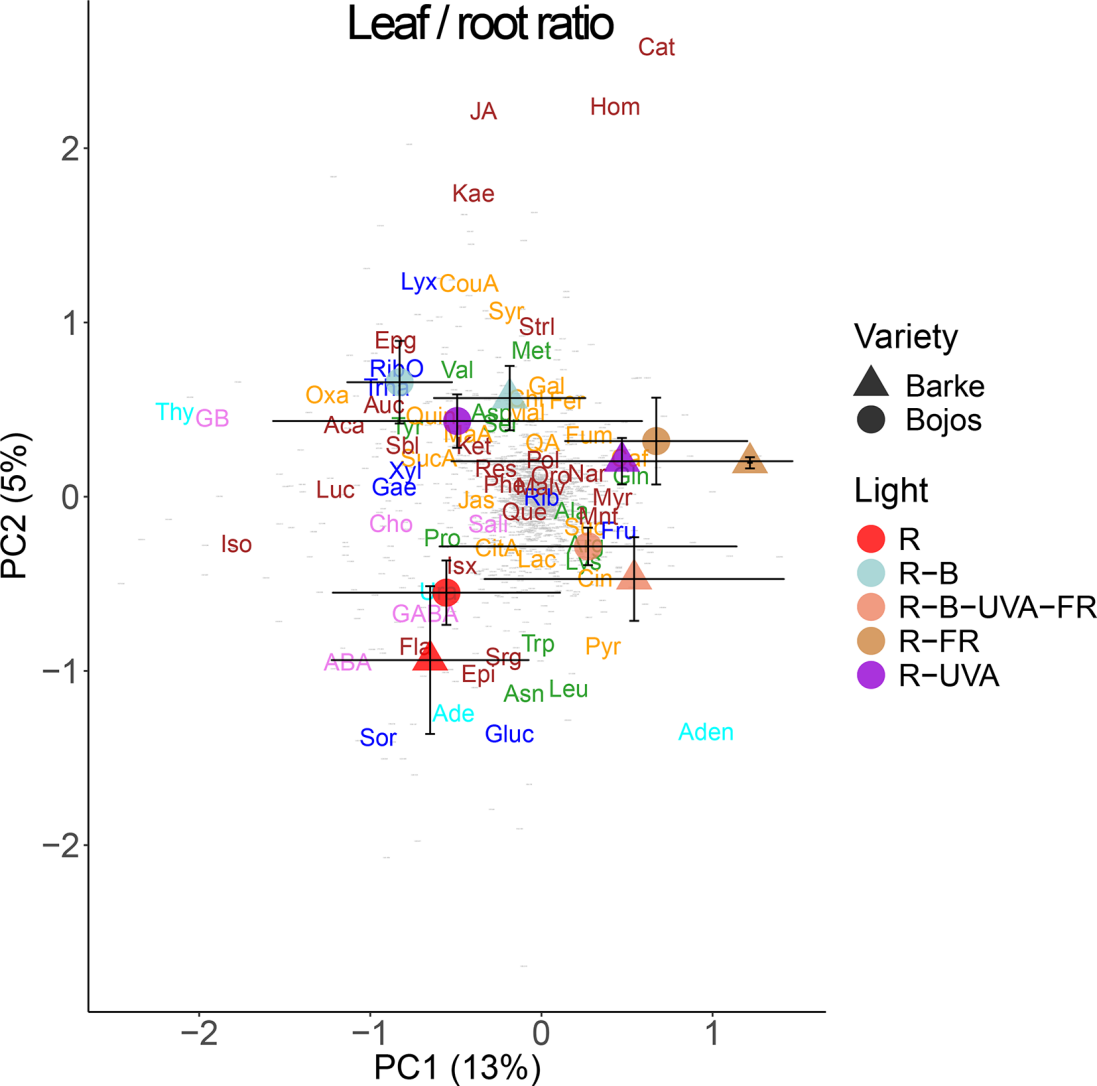
Principal component analysis (PC1 *vs.* PC2) of root-to-leaf ratios of metabolite concentrations of two barley varieties grown for 26 days under different light regimens. Additional details are in the **Figure 6** legend.

We also analyzed the changes in metabolite allocation between leaves and roots, expressed as leaf:root ratios (**Figure 9**). PC1 mainly separated the effects of the R-FR and R-B-UVA-FR regimens. In particular, these treatments were mainly associated with more allocation of fructose, myricetin, naringenin, mannitol, and glutathione to roots than leaves. PC2 mainly separated the effects of the R-B and R-UVA regimens, which were associated with more allocation of JA, kaempferol, *p*-coumaric acid, and D-lyxose to roots than leaves. On the other hand, the R regimen was associated with more allocation of glucose, sorbitol, and ABA to roots than leaves.

**Figure 10:**
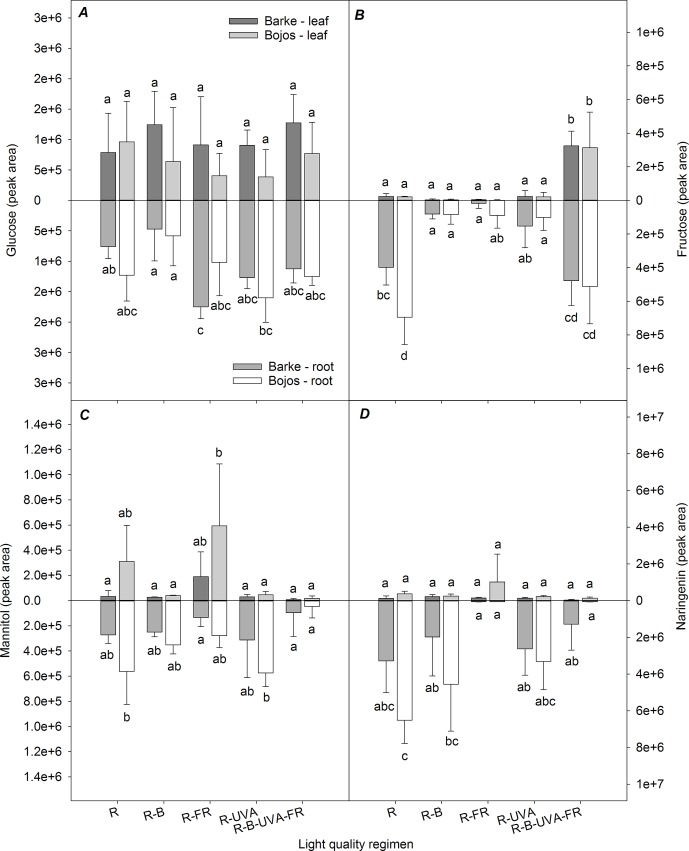
Effect of 26 days exposure to different light regimens on allocation of selected sugars (glucose, **A**; fructose, **B**; mannitol, **C**) and a selected phenolic compound (naringenin, **D**) to leaves and roots of spring barley varieties Barke (dark grey) and Bojos (light grey). Additional details are in the **Figure 1** legend.

**Figure 10** shows the selected metabolites that had large differences in roots and root/leaf ratios among the different light regimens. Although the glucose content had large variance in roots and leaves, the R-FR regimen (Barke variety) and the R-UVA regimen (Bojos variety) altered the leaf:root ratios (**Figure 10A**). Relative to the R regimen, leaf fructose level was significantly greater under the R-B-UVA-FR regimen (by 1,371%) but lower under the R-B and R-FR (by 86% and 87%, respectively), and this led to significant effects on the leaf:root ratio (**Figure 10B**). The light regimen also had a significant effect on the leaf:root ratio for mannitol, with a particularly high ratio under the R-FR regimen and a decreased ratio under the R-UVA regimen (**Figure 10C**). Similarly, root naringenin was only present at very low levels under the R-FR and R-B-UVA-FR regimens (**Figure 10D**).

## Discussion

### Responses to Far-Red Light

Phytochromes in a plant shoot sense changes in the R:FR ratio and signal that neighboring plants are competing for light. This signal allows competing plants rapid elongation of leaves and stems and upward reorientation of leaves (Franklin, 2008). In monocotyledonous plants, a low R:FR ratio also stimulates apical dominance and reduces tiller formation (Kebrom and Brutnell, 2007). In accordance with these results, we found that the two varieties of barley studied here had significant increases of leaf length and TLA under the R-FR regimen relative to the R regimen.

Less is known about the effect of the R:FR ratio on root development. However, there is evidence that a low R:FR ratio leads to a reduced root-to-shoot biomass ratio in soybean (Kasperbauer, 1987), reduced lateral root density in *Arabidopsis thaliana* (Gelderen van et al., 2018b), and reduced root hair density in *Arabidopsis thaliana* (de Simone et al., 2000). However, these findings differ from our results for barley, which showed that a low R:FR ratio stimulated most RSA traits, although it reduced root BA. Lee et al. (2015) showed that the response of root biomass to the R:FR ratio is non-linear and that decreasing the R:FR ratio beyond a certain level stimulated the growth of root biomass and increased the root-to-shoot ratio. An interactive effect of circadian clock and R:FR ratio on root and shoot growth mediated by phytochrome-interacting factor 4 (PIF4) and PIF5 transcription factors also has to be taken into account. While the PIF4 and PIF5 expression peaks in the morning hours of long days, the maximum expression occurs in the night when the photoperiods are short (Farré, 2012).

Light-induced regulation of plant growth, physiology, and metabolism involves multiple hormonal pathways such as gibberellins, ABA, auxins, ethylene, and cytokinins; however, the molecular links between these pathways and light signaling are not yet completely understood (Lau and Deng, 2010). A low R:FR ratio stimulates the accumulation of at least three plant hormones: gibberellins (de Lucas et al., 2008), auxin (Procko et al., 2014), and ethylene (Kurepin et al., 2010). A low R:FR ratio also desensitizes plants to defense-associated plant hormones, such as JA and salicylic acid. Auxin is the dominant physiological regulator when plants are growing in an environment with a low R:FR ratio (Yang and Li, 2017), and its polar transport affects root growth and development (Saini et al., 2013). The complex nature of auxin shoot-to-root transport and its interactions with other plant hormones may lead to a wide range of shoot and root responses (Lavenus et al., 2013), as observed under the different light regimens used in this study. Moreover, a recent study showed that JA, whose abundance was affected by the R:FR ratio, links the molecular mechanisms responsible for plant growth and immunity (Campos et al., 2016). This suggests that the R:FR ratio determines the allocation of plant resources to growth or defense. However, our results showed that FR light had only a small effect on JA biosynthesis in leaves, in contrast to B light.

Previous research indicated that JA (Suzuki et al., 2011) and photosynthetic products (Kircher and Schopfer, 2012) might link the detection of the R:FR ratio by the shoot with morphogenesis in the root. In accordance with other research (Park and Runkle, 2017), we found that the presence of FR stimulated photosynthesis. Nevertheless, our metabolomic analyses indicated an increased content of glucose in roots and reduced content in leaves in the presence of FR. A high glucose content can affect root length, a number of lateral roots, and root growth direction because of the strong interactions of glucose with auxin (Mishra et al., 2009). On the contrary, we found that fructose and mannitol levels were reduced in roots under the R-FR regimen, which indicates that different sugars have different effects on root growth. Although the function of fructose as sugar regulator is less explored, its interactions with signaling pathways of ABA and ethylene suggest an inhibitory role in root growth (Cho and Yoo, 2011). In contrast, sucrose promotes cell division in root meristems, modifies the expression of numerous genes related to P deficiency, and thus leads to altered root growth and morphology (Horacio and Martinez-Noel, 2013). On the other hand, mannitol and other sugar alcohols play a role in reactive oxygen species (ROS) scavenging rather than in plant growth regulation (Bolouri-Moghaddam et al., 2010).

### Responses to Blue Light

Blue light is an essential environmental signal that also controls plant cell elongation (Huché-Thélier et al., 2016). Blue light represses gibberellin synthesis and auxin synthesis and affects several genes involved in growth repression (Folta et al., 2003). Although the “shade avoidance syndrome” is most often associated with a reduced R:FR ratio, some studies have shown that a reduced level of blue light had a similar effect and suggested that a PHY-B–independent hormonal cascade can also lead to the shade avoidance syndrome (Keller et al., 2011). The blue light–induced shade avoidance syndrome is predominantly mediated by CRY1 and brassinosteroid hormones. Previous studies showed that a high blue-to-red light ratio increased biomass in wheat (Goins et al., 1997) but decreased biomass in lettuce (Kang et al., 2016) and rice (Ohashi-Kaneko et al., 2006). Folta and Childers (2008) reported that responses to blue light are organ-specific and depend on the total photon flux density and blue-to-red light ratio. In our study, the R regimen and the R-B regimen had similar effects, in that the R-B regimen only significantly increased leaf length in the Bojos variety. The blue light also had no significant effect on RSA parameters in either variety of barley.

Many studies demonstrated that supplementary blue light increased photosynthetic performance (Hogewoning et al., 2010; Wang et al., 2016), chlorophyll (Chl) content, and the Chl *a*/*b* ratio (Hogewoning et al., 2010; Kopsell and Sams, 2013). Similarly, we found that the addition of blue light (R-B regimen) significantly increased most of the photosynthetic parameters in our two barley varieties. An increased CO_2_ assimilation rate under blue light is particularly associated with increased stomatal conductance (Boccalandro et al., 2012) being mediated by phototropins PHOT1 and PHOT2 (Kinoshita et al., 2001). However, high photosynthetic carbon uptake may lead, under the conditions of limited C sink, to the accumulation of soluble sugars and consequently to stomata closure. Such feedback regulation is mediated by hexokinase within the guard cells of stomatal pores (Granot and Kelly, 2019). If there is sufficient sink for sugars in roots, these (mainly sucrose) are rapidly transported by phloem and stimulate root growth, the process mediated by auxin and cytokinin (Wang and Ruan, 2016). Re-allocation of sugars between roots and shoots is tightly coupled with plant N metabolism. N uptake controls the availability of sugars because carbon skeletons are essential for the assimilation of inorganic N into amino acids, proteins, and nucleic acids and vice versa. On the other hand, N-deficiency reduces the rates of photosynthetic carbon uptake and accumulation of sugars (Wang and Ruan, 2016).

Several studies, however, reported that photosynthetic performance increased with increasing blue light intensity only in the presence of other wavelengths (Hogewoning et al., 2010; Hernández and Kubota, 2016). The opposite effects of blue light on growth and photosynthesis can be the reason for observed discrepancies in biomass formation under blue light conditions. The role of blue light signaling in RSA is still not well understood. However, under blue light, *cry* mutants of *Arabidopsis* have substantial differences in primary root length relative to wild type (Canamero et al., 2006).

In our study, the R-B regimen (relative to the R regimen) led to a reduced content of ABA and an increased content of JA in leaves. This is in accordance with stomatal responses to the R-B regimen. Boccalandro et al. (2012) examined the roles of CRY1, CRY2, and ABA in stomatal regulation and reported that blue light led to a substantial increase of stomatal conductance and a reduced ABA content, but this did not occur in CRY1 or CRY2 mutants.

Some studies suggested a close relationship between the biosynthesis of ABA and JA (Brossa et al., 2011). However, these results may simply indicate that reduced water availability independently stimulates JA and ABA accumulation. Previous studies have examined the nature of this relationship using mutants. In particular, although the blue light–induced reduction of ABA content is mediated by CRY1 and CRY2, blue light also increases JA biosynthesis (Svyatyna and Riemann, 2012). Blue light stimulates the biosynthesis of JA by activating the expression of genes involved in JA biosynthesis, although phytochrome A signaling has a stronger effect. Although mechanistic studies with phytochrome mutants reported a crucial role of the R:FR ratio in regulation of the JA biosynthesis and resistance to fungal pathogens (Cerrudo et al., 2012), our results showed that supplementary blue light significantly increased JA accumulation, and this could provide increased resistance to environmental stressors. Blue light also induced the accumulation of several secondary metabolites in barley varieties studied, such as *p*-coumaric acid, chlorogenic acid, aucubin, and homoorientin (but had no impact on isovitexin or kaempferol), which increased in plants receiving the R-B-UVA-FR regimen. Generally, these secondary metabolites play important roles as antioxidants scavenging ROS, UV screening molecules, or anti-infection agents (reviewed by Falcone Ferreyra et al., 2012). Their role also depends on location within the leaf, having screening functions when located in the adaxial epidermis and antioxidant functions when located in the mesophyll. This is particularly true for dihydroxy B-ring–substituted flavonols such as kaempferol or quercetin (Agati et al., 2012). Soluble hydroxycinnamates such as *p*-coumaric acid or chlorogenic acid and mesophyll flavonoids are constitutively present in young leaves, but these are gradually replaced by epidermal flavonols in mature leaves acclimated to light conditions (Burchard et al., 2000; Agati et al., 2013). Walter et al. (2010) summarized the role of cell wall–associated plant secondary metabolites, such as *p*-coumaric acid and other hydroxycinnamic acids, in the inhibition of fungal growth and resistance to *Fusarium* head blight infection. Their results indicated that the blue light–induced accumulation of JA and some phenolic compounds had an important role in the blue light–induced resistance to fungal infection.

We found that blue light strongly stimulated the accumulation of the sesquiterpene sorgolactone, a strigolactone that stimulates germination of parasitic weeds. However, strigolactones also act as endogenous hormones that regulate shoot branching, secondary growth of stems, leaf senescence, primary root growth, adventitious root formation, and root hair elongation (Seto et al., 2012). Strigolactones also increase plant tolerance to drought and salinity, mainly through its effect on stomatal density and responsivity (Ha et al., 2014). Additional studies are, however, needed to further elucidate the effect of blue light on this regulatory pathway.

### Responses to UV-A Radiation

PHOTs and CRYs both percept UV-A and blue light (Gyula et al., 2003), so activation of these photoreceptors may be expected to have similar effects on hormone production. Our results, however, show that UV-A radiation did not have the same effect as the blue light on CO_2_ assimilation and stomatal opening. Also, the R-UVA regimen had a weaker effect on TLA and root parameters than the R-B regimen.

Recently, we have also shown that UV radiation alone, and in combination with elevated atmospheric CO_2_ concentration, alters N allocation between leaves and roots of European beech saplings, leading to changes in leaf and root C:N stoichiometry and morphological traits (Uchytilová et al., 2019). C:N stoichiometry can be considered as one of the most relevant indicators of source:sink balance and can regulate expression of several genes involved in N metabolism (Nunes-Nesi et al., 2010), secondary metabolism (Royer et al., 2013), photosynthesis (Ruiz-Vera et al., 2017; Foyer et al., 2018), and plant biomass allocation (Kruse et al., 2010).

Several studies reported that supplemental UV-A radiation increases leaf area (Biswas and Jansen, 2012; Zhang et al., 2014). However, UV-A radiation probably acts with other signaling pathways to increase the elongation of different leaf parts, suggesting the presence of intercellular signaling (Verdaguer et al., 2017). Some studies have also shown the higher allocation of resources to root biomass under UV-A radiation (Zhang et al., 2014), but others reported that UV-A radiation had a negative effect or no effect on root biomass (Cooley et al., 2001).

Although UV-B radiation has a major role in the induction of flavonoid biosynthesis, UV-A radiation also promotes the accumulation of flavonoids in many species (Morales et al., 2010). However, rather than total phenolics, UV-A radiation regulates the accumulation of specific phenolic compounds (Verdaguer et al., 2017). While UV-A radiation had only a minor effect on the accumulation of flavonoids in our study, the R-B-UVA-FR regimen induced accumulation of most flavonoids, indicating that various spectral bands may contribute to flavonoid biosynthesis. Similarly, our earlier study has proven the fundamental importance of the combined effect of PAR and UV on the accumulation of phenolics such as lutonarin and 3-feruloylquinic acid, xanthophyll cycle pigments, and subsequent tolerance to high radiation stress (Klem et al., 2015).

UV-A radiation can also ameliorate the UV-B radiation–induced damage to DNA by promoting DNA repair (Krizek, 2004). On the other hand, several studies reported that UV-A radiation damages PSII (Nawkar et al., 2013), although we observed no such effect. Under natural conditions, UV-A radiation accounts for most of the sunlight-induced damage to photosynthesis because there is much more UV-A than UV-B radiation in the solar spectrum (Verdaguer et al., 2017).

One of the compounds whose level increased the most by UV-A radiation in our study was GABA (a non-protein amino acid). This compound rapidly accumulates in plant tissues in response to biotic and abiotic stress and is a putative signaling molecule (Roberts, 2007). GABA may also have possible roles in the interfacing of C and N metabolism (Michaeli and Fromm, 2015) and protection against oxidative stress and herbivorous pests (Bouché and Fromm, 2004). Other studies reported that increased GABA levels correlated with leaf senescence (Diaz et al., 2005) and that GABA responses depend on ethylene and ABA signaling (Lancien and Roberts, 2006). Mekonnen et al. (2016) used a GABA-depleted mutant of *Arabidopsis thaliana* to demonstrate the role of this compound in stomatal regulation and sensitivity to drought stress. Li et al. (2013) suggested that the effect of light on GABA accumulation is due to light-induced upregulation of glutamate decarboxylase or glutamic acid decarboxylase (GAD). To our best knowledge, the present study is the first to show that UV-A radiation increases the biosynthesis of GABA.

### Interactions of UV-A, Blue, and Far-Red Light

The R-B-UVA-FR regimen had the strongest effects on increasing leaf area and photosynthetic parameters. This suggests the importance of simultaneous irradiation with multiple regions of the spectrum. The effect of the R-B-UVA-FR regimen on photosynthetic parameters was comparable to the R-B regimen, suggesting that blue light had a greater effect than FR or UV-A light on this variable. Relative to the R regimen, the R-B-UVA-FR regimen strongly increased the above-ground biomass but had negligible effects on root biomass and root architecture parameters. This suggests that the addition of blue light, UV-A radiation, or both impaired the positive effect of FR light on the growth of seminal and lateral roots.

Relative to the R regimen, the R-B-UVA-FR regimen also increased the accumulation of specific amino acids (proline, valine, leucine), phenolics (isovitexin, *p*-coumaric acid, kaempferol), and saccharides (fructose) in leaves (**Figures 6**, **7**, and **9**). Moreover, such changes under the R-B-UVA-FR regimen resulted in significant increases in leaf:root ratio of saccharides (particularly fructose), which were low in other regimens. This important result indicates that light can induce changes in the transport of saccharides from the shoots to the roots.

A previous study showed that sucrose activated and promoted root elongation (Kircher and Schopfer, 2012), and coordinated root and shoot growth with light intensity and spectral quality (Wang and Ruan, 2016). Sugars also provide crosstalk with other signaling and metabolic processes to regulate local and systemic signaling pathways (Ljung et al., 2015), which is integrated *via* PIF (Stewart et al., 2011). However, as discussed earlier, the roles of individual sugars can be different as glucose and sucrose interact mainly with auxin and promote plant growth, while fructose interacts with inhibiting plant hormones such as ABA or ethylene.

It has been demonstrated by Ahanger et al. (2018) that accumulation of osmolytes, antioxidants, carotenoids, and other defense compounds under environmental stress exhibits crosstalk with the cascade of phytohormone signaling, which is mediated by brassinosteroids. Brassinosteroids can have synergistic or antagonistic interactions with other phytohormones for elicitation of stress response involving the antioxidant system, synthesis osmotic constituents, or other mechanisms. Thus, brassinosteroids play a potentially integrating role in responding to light quality and inducing plant protection mechanisms for stress.

## Conclusion

Our findings suggest that light quality has a different impact on the shoot and root morphology, physiology, and biochemistry, which may have major implications for plant performance under stress conditions. While the growth of above-ground biomass and photosynthetic performance were enhanced mainly by the combined action of red, blue, far-red, and UV-A light, the root growth was stimulated particularly by supplementary far-red light to red light. These findings may have significant implications for improving root development and, consequently, the drought resistance, e.g., by adjusting sowing density. Exposition of plants to the full light spectrum stimulates the accumulation of defense compounds such as proline, secondary metabolites with antioxidative functions, or JA, and reduces the accumulation of ABA, which allows to assume increased stress tolerance. In addition, blue light induced accumulation of GABA, sorgolactone, or several secondary metabolites. As these compounds play important roles as osmolytes, antioxidants, UV screening compounds, or growth regulators, the importance of light quality in the induction of protective mechanisms against abiotic and biotic stress factors is unequivocal. Application of a suitable spectrum of light wavelengths by specific plastic filters, monochromatic LED light sources, and/or crop management measures may thus provide important tools for adjusting plant tolerance to abiotic and biotic stress.

## Data Availability

The datasets generated for this study are available on request to the corresponding author.

## Author Contributions

KK, OU, and WR designed the experiments. KK, WR, BV, and PH conducted the experiment, MO conducted the metabolomic analyses, WR conducted the root system architecture analysis, and AG-G and KK conducted the statistical analyses of data. KK, AG-G, PH, JS, JP, and OU wrote the manuscript. All authors read and approved the final manuscript.

## Funding

This research was supported by SustES—Adaptation strategies for sustainable ecosystem services and food security under adverse environmental conditions, project no. CZ.02.1.01/0.0/0.0/16_019/0000797.

## Conflict of Interest Statement

The authors declare that the research was conducted in the absence of any commercial or financial relationships that could be construed as a potential conflict of interest.

## Supplementary Material

Figure S1 Scheme of the growth unit layout (upper right), growth unit with the growing plant (upper left), and arrangement of growth units in a plastic bin with micro-irrigation pump inside.
