# Supplementary figures and images for: Distinct Morphological, Physiological, and Biochemical Responses to Light Quality in Barley Leaves and Roots
Source: Front Plant Sci. 2019 Aug 14;10:1026. doi: 10.3389/fpls.2019.01026 (PMC6703096; doi:10.3389/fpls.2019.01026)

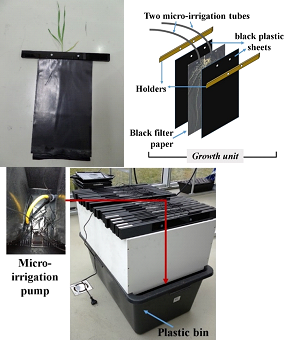

Supplement: Figure S1 — Scheme of the growth unit layout (upper right), growth unit with the growing plant (upper left), and arrangement of growth units in a plastic bin with micro-irrigation pump inside. [file Image_1.tif]
